# Cardiac-Restricted IGF-1Ea Overexpression Reduces the Early Accumulation of Inflammatory Myeloid Cells and Mediates Expression of Extracellular Matrix Remodelling Genes after Myocardial Infarction

**DOI:** 10.1155/2015/484357

**Published:** 2015-09-30

**Authors:** Enrique Gallego-Colon, Robert D. Sampson, Susanne Sattler, Michael D. Schneider, Nadia Rosenthal, Joanne Tonkin

**Affiliations:** ^1^National Heart and Lung Institute, Imperial College London, London, UK; ^2^Australian Regenerative Medicine Institute, EMBL Australia, Monash University, Clayton, Melbourne, VIC, Australia; ^3^The Jackson Laboratory, Bar Harbor, ME, USA

## Abstract

Strategies to limit damage and improve repair after myocardial infarct remain a major therapeutic goal in cardiology. Our previous studies have shown that constitutive expression of a locally acting insulin-like growth factor-1 Ea (IGF-1Ea) propeptide promotes functional restoration after cardiac injury associated with decreased scar formation. In the current study, we investigated the underlying molecular and cellular mechanisms behind the enhanced functional recovery. We observed improved cardiac function in mice overexpressing cardiac-specific IGF-1Ea as early as day 7 after myocardial infarction. Analysis of gene transcription revealed that supplemental IGF-1Ea regulated expression of key metalloproteinases (MMP-2 and MMP-9), their inhibitors (TIMP-1 and TIMP-2), and collagen types (Col 1*α*1 and Col 1*α*3) in the first week after injury. Infiltration of inflammatory cells, which direct the remodelling process, was also altered; in particular there was a notable reduction in inflammatory Ly6C+ monocytes at day 3 and an increase in anti-inflammatory CD206+ macrophages at day 7. Taken together, these results indicate that the IGF-1Ea transgene shifts the balance of innate immune cell populations early after infarction, favouring a reduction in inflammatory myeloid cells. This correlates with reduced extracellular matrix remodelling and changes in collagen composition that may confer enhanced scar elasticity and improved cardiac function.

## 1. Introduction

Cardiovascular diseases (CVD) are the major cause of death globally, with myocardial infarction (MI) being one of the main causes of mortality [1]. After MI, the damaged myocardium releases inflammatory signals that trigger a cascade of cellular processes in order to repair damaged tissue, leading to the formation of scar tissue and left ventricular (LV) dysfunction [2, 3].

Our laboratory has characterised the therapeutic properties of the insulin-like growth factor-1 Ea (IGF-1Ea) propeptide during wound healing/regeneration and pathological inflammation. The* IGF-1* gene is encoded in 70 kb of genomic DNA distributed over six exons and five introns [4, 5]. Use of alternative start codons generates proteins with N-terminal variability while different exon use at the 3′ end generates multiple C-terminal extension-peptides, termed E-peptides. The most predominant is a 35-amino-acid-long E-peptide, termed Ea, alternating with a far less abundant E-peptide termed Eb or mechanogrowth factor (MGF) [6, 7]. The E-peptides control local IGF-1 bioavailability by adhering strongly to the extracellular matrix (ECM), retaining the propeptides locally and preventing their release into the circulation [8]. Expressed as a cardiomyocyte-specific transgene or delivered locally to the mouse heart, IGF-1Ea improves functional recovery after cardiac injury [9, 10]; however the underlying mechanisms are not fully understood.

Tissue restructuring after infarction involves the breakdown of the ECM by proteolytic enzymes, mainly the matrix metalloproteinases (MMP) MMP-2 and MMP-9, balanced by interaction with tissue inhibitors of metalloproteinases (TIMPs) [11]. Initially a temporary matrix is formed, primarily composed of collagen type III (Col I*α*3), providing a scaffold for replacement cells and structural integrity to the heart, thereby reducing the risk of LV dilation and rupture [12, 13]. This is later replaced by collagen type I (Col I*α*1) which will constitute the permanent ECM [14]. Col I*α*1 confers tensile strength and resistance to stretch and deformation, while Col I*α*3 confers compliance. Their balance determines cardiac tissue stiffness with increased Col I*α*3 to Col I*α*1 ratio generating a more resilient left ventricle [13, 14].

Innate immune cells recruited to the injured myocardium from the blood include neutrophils, monocytes, macrophages, and dendritic cells [15–17]. They play a prominent role in remodelling, producing the MMPs that break down the matrix, synthesising new ECM components, and activating fibroblasts to myofibroblasts which will later in the inflammatory process be the main producers of matrix proteins [14]. The tissue microenvironment at a given time after MI influences the recruitment of immune cells as well as their phenotypic and functional properties. This is especially relevant for the macrophage population which undergoes a time-dependent shift between inflammatory and reparative functions [3, 18]. At early time points after injury, the majority of macrophages produce inflammatory cytokines and reactive oxygen species including interleukin- (IL-) 1*β*, IL-12, MMP-9, and nitric oxide. These are termed inflammatory or M1-polarised macrophages, which express high levels of Ly6C and function to recruit more inflammatory cells and phagocytose cellular debris and produce growth factors [12]. As the inflammation progresses, reparative macrophages accumulate in the infarct area. In contrast to the inflammatory macrophages, these cells, many of which express CD206, are involved in the suppression of inflammation due to high production of IL-10 and TGF-*β* [3, 19] and assist in the progression from inflammation to repair.

They also perform reparative roles promoting cell growth, angiogenesis, and remodelling of the ECM. Additionally, different monocyte populations can be distinguished by Ly6C in the mouse and may preferentially give rise to inflammatory versus reparative macrophages.

We have previously shown that IGF-1Ea and its mature circulating form IGF-1 can modulate immune responses and suppress pathological inflammation by inducing regulatory cytokines and immune cell types [20, 21]. In the heart, IGF-1Ea increased expression of IL-10 after cardiotoxin injury and decreased levels of IL-1*β* suggesting that a shift in immune cell populations may also occur in the heart [9]. In the present study, we investigated whether there was a difference in the immune cell dynamics after MI in transgenic IGF-1Ea hearts and whether this had a carry-on effect on tissue remodelling.

## 2. Materials and Methods

### 2.1. Cardiac Injury Model

Myocardial infarction by permanent left coronary artery occlusion was induced in wild-type (WT) and *α*MHC.IGF-1Ea male mice which were 8 to 12 weeks old. Surgeries were performed under mechanical ventilation with 1–2.5% isofluorane. The chest cavity was opened through the left fourth intercostal space. The heart was exposed and the left coronary artery was ligated using an 8.0 mm non-absorbable suture (Ethicon-Johnson  &  Johnson, USA) below the left atrium to produce an ischemic region of 20–30% of the left ventricle area. The chest cavity and skin were sutured with 6.0 mm silk sutures (Ethicon-Johnson & Johnson, USA). Analgesic treatment with buprenorphine (0.3 mg/kg, s.c.) was provided before and after surgery. They were housed in individually ventilated cages in temperature-controlled facilities on a 12-hour light/dark cycle on standard diet. All mouse procedures were approved by the Imperial College London Ethical Committee and were in accordance with national and international regulations (UK Home Office Project license 70/7589).

### 2.2. Echocardiography

Echocardiographic measurements were taken using a high-frequency ultrasound system Vevo 770 (VisualSonics, Inc., Canada) with a 30 MHz linear transducer and recorded images were analyzed by using the Vevo 770 workstation software. Mice were anesthetised with 1-2% isofluorane, and the anesthetic flow rate was adjusted to maintain heart rate of approximately 450 ± 50 beats per minute. Furthermore, warmed ultrasound gel and a heating platform were used to maintain body temperature at 37 ± 0.5°C to minimise variation between mice. This analysis was performed at basal level, 1, 3, 4, 7, and 28 days after MI to evaluate left ventricle cardiac function, chamber dimensions, and infarct size.

### 2.3. Masson's Trichrome Staining

Samples were fixed in paraformaldehyde (4% in PBS) for 48 h, washed in PBS, dehydrated, and embedded in paraffin wax. Five-micron-thick sections were stained with Celestine Blue for 5 minutes, washed in tap water, and then incubated in haematoxylin for 5 minutes. Slides were then incubated with Acid Fuchsin for 5 minutes, rinsed in distilled water, incubated in phosphomolybdic acid (1%), and then rinsed in distilled water before staining with Methyl Blue for 2-3 minutes. Slides were dehydrated in ascending concentrations of ethanol, cleared in xylene, and mounted in DPX (VWR, UK).

### 2.4. Cell Isolation

To analyse neutrophils, monocytes, macrophages, and dendritic cells, a single cell suspension was prepared from hearts before or at various time points after MI (days 1, 3, 5, 7, and 28 after operation). The hearts were mechanically dissociated using surgical scissors and subsequently treated with a 1x Hank's Balanced Salt Solution (HBSS) (Invitrogen, USA), enzymatic dissociation buffer containing 0.1 mg/mL Liberase TH Research Grade (Roche Diagnostics, UK), 50 *μ*g/mL of DNaseI (Roche Diagnostics, UK), 10 mM HEPES (Invitrogen, USA), and 30 mM Taurine (Sigma, UK) for 4 cycles of 10 min at 37°C. After each 10 min incubation cycle, the cells were collected and filtered using a 70 *μ*m cell strainer (BD Pharmingen, USA) and an equal volume of ice cold 1x HBSS containing 10 mM HEPES, 30 mM Taurine (Sigma, UK), and 20% Fetal Bovine Serum (FBS) (GE Healthcare, USA) was added to the enzyme dissociation buffer. The cells were pelleted at 320 g for 7 min at 4°C and washed with the 1x HBSS media solution containing 20% FBS as described above. Under these isolation conditions, adult cardiomyocytes are predominately lysed, as the enzymatic dissociation buffer is toxic to these large, fragile cells [22]. An aliquot of the cell suspension was used to quantify the cell concentration/mL using a Beckman Coulter Vi-Cell XR cell counter (Beckman Coulter, High Wycombe, UK).

### 2.5. Flow Cytometry and Cell Sorting

Isolated cells were incubated in a 1x Dulbecco's Modified Eagle Medium (DMEM) solution (Gibco, Life Technologies, UK) containing 2% FBS and 10 mM HEPES for 30 min, on ice, in the dark, with the following primary antibodies: CD11b-PE (BD Biosciences; catalogue 553311), F4-80-Biotin (eBioscience; catalogue 13-4801-85), CD45-APC-Cy7 (BioLegend; catalogue 103116), CD206-PerCP-Cy5.5 (BioLegend; catalogue 141716), Ly6C-APC (eBioscience; catalogue 17-5932-82), and Ly6G-AlexaFluor700 (BioLegend; catalogue 127622). Samples stained with biotin-labelled primary antibody were incubated with a streptavidin-PE-Cy7 (eBioscience; catalogue 25-4317-82) secondary antibody for 30 min, on ice, in the dark, with the 1x DMEM media solution as mentioned above. The samples were washed and resuspended in fresh 1x DMEM media solution with 1.5 *μ*M Sytox Blue (Invitrogen. USA) dead cell stain and refiltered using 5 mL, 35 *μ*m filter cap tubes (BD Falcon) just prior to sample acquisition. Flow cytometric cell sorting was performed using a BD FACSAriaI cell sorter (BD Biosciences, Oxford, UK) equipped with a 355 nm UV laser, a 405 nm Violet laser, a 488 nm Blue laser, a 561 nm Yellow-Green laser, and a 640 nm Red laser. The antibody cocktail fluorescence minus one (FMO) controls were used as gating controls for analyses to distinguish positive from negative fluorescence signal (see Supplementary 4–6 in Supplementary Material available online at http://dx.doi.org/10.1155/2015/484357). Total leukocytes (CD45+), neutrophils (CD45+, CD11b+, F4/80−,   CD11c−, and Ly6G+), and monocytes (CD45+, CD11b+, CD11c−, Ly6G−, and F4/80−) were analysed. Monocytes were further classified as Ly6C^high^ and Ly6C^low^ monocytes. Macrophages were defined as CD45+, CD11b+, CD11c−, Ly6G−, and F4/80+ and further characterised on the basis of Ly6C and CD206 expression (i.e., Ly6C^high^/CD206^low^ (inflammatory macrophages) and Ly6C^low^/CD206^high^ (reparative macrophages)). Dendritic cells were defined as CD45+, CD11b+, F4/80−, CD11c+, and Ly6G−. Flow Jo software (version 9. 8.5) (Tree Star, Ashland, OR) was used for analysis.

### 2.6. RNA Isolation and cDNA Generation

Mouse heart tissue was harvested and flushed with cold PBS. Samples from the infarct and remote (non-infarct) myocardium were placed in a 1.5 mL tube and homogenised in TRIzol (Invitrogen, USA) reagent using a rotor-stator homogeniser (Polytron PT 2500 E). Total RNA was isolated according to the TRIzol manufacturer's instructions. RNA was pelleted, air-dried, and resuspended in DNase/RNase free water and the yield quantified using Nanodrop (Thermo Scientific, USA) at 260 nm. One microgram of RNA was reverse-transcribed into cDNA using the Quantitect Reverse Transcription Kit (QIAGEN, Crawley, UK).

### 2.7. Quantitative Real-Time PCR

Following cDNA synthesis, quantitative RT-PCR using TaqMan probes (Invitrogen. USA) was performed on an ABI 7900HT Sequence Detection System (Applied Biosystems, Carlsbad, CA, USA). The probes used were IGF-1Ea (Mm00710307_m1), IL-10 (Mm00439614_m1), IL-1*β* (Mm00434228_m1), CCL2 (Mm00441242_m1), CCL5 (Mm01302427_m1), TGF*β* (Mm03024053_m1), collagen I*α*1 (Mm00801666_g1), collagen I*α*3 (Mm01254476_m1), Lox (Mm00495386_m1), MMP2 (Mm00439498_m1), MMP9 (Mm00442991_m1), TIMP1 (Mm00441818_m1), TIMP2 (Mm004418225_m1), actin, alpha 1, skeletal muscle, ACTA, (Mm00808218_g1), Atrial Natriuretic Peptide, and ANP (Mm01255747_g1). Gene expression was determined as fold induction over uninjured hearts after normalising to the reference gene, GAPDH.

### 2.8. Statistics

Data are presented as mean ± SEM. Two-tailed Student's* t*-test was performed to compare WT and *α*MHC.IGF-1Ea mice at selected time points after MI. Data were analysed with GraphPad-Prism 5.0 (Graphpad Software, Inc., www.graphpad.com), and differences were considered statistically significant at *P* < 0.05.

## 3. Results

### 3.1. IGF-1Ea Improves Cardiac Function after Myocardial Infarction

**Figure 1:**
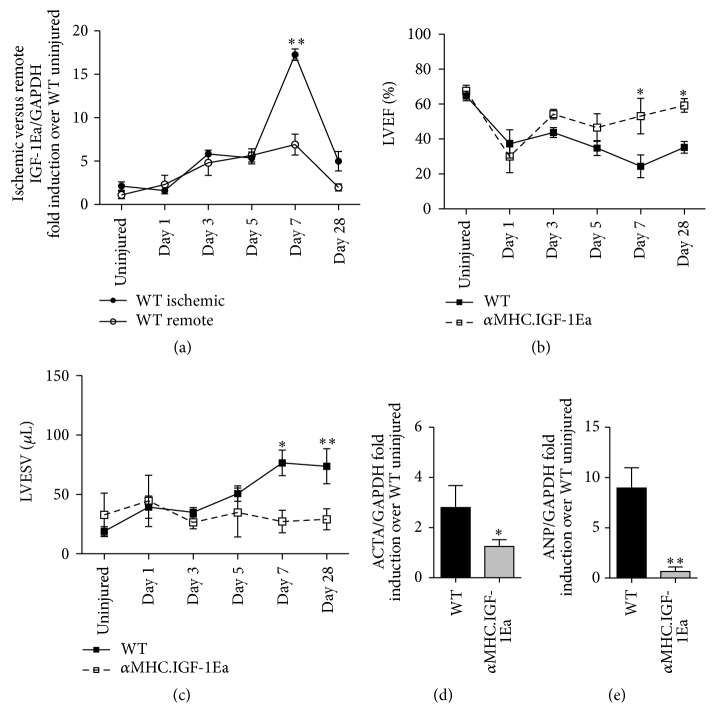
*α*MHC.IGF-1Ea improves cardiac function and reduces dilation as early as 7 days after myocardial infarction. (a) Levels of IGF-1Ea in the ischemic and in the remote area of WT hearts at 1, 3, 5, 7, and 28 days following MI. (b) Left ventricular ejection fraction (LVEF) and (c) left ventricular end systolic volume (LVESV) after MI. Solid lines represent WT mice. Dashed lines represent *α*MHC.IGF-1Ea mice. Levels of (d) actin-alpha 1 skeletal muscle (ACTA 1) and (e) atrial natriuretic peptide (ANP) mRNA expression 28 days after MI. Results are expressed as mean fold induction ± SEM over the values of uninjured hearts. *n* = 4–6 per group. Two-tailed Student's* t*-test was performed to compare WT versus *α*MHC.IGF-1Ea at selected time points after MI. ^*∗*^ *P* < 0.05, ^*∗∗*^ *P* < 0.005.

Endogenous IGF-1Ea expression in WT hearts was measured 1, 3, 5, 7, and 28 days after MI in both ischemic and remote (nonischemic) regions. IGF-1Ea levels increased in both the ischemic and remote regions (Figure 1(a)) with a substantially stronger induction in the ischemic area (8-fold over uninjured levels). These results indicate that, similar to other organs, endogenous IGF1-Ea expression is upregulated after cardiac tissue damage [23, 24]. In *α*MHC.IGF-1Ea hearts, the baseline expression of transgenic IGF-1Ea was much higher than the expression of endogenous IGF-1Ea in WT hearts (average 286-fold) at all experimental time points (Supplementary 1A and 1B). Although far exceeding the expression of endogenous IGF-1Ea, the *α*MHC.IGF-1Ea transgenic mice provide a suitable model of IGF-1Ea at supraphysiological concentrations of possible therapeutic relevance.

Previously our group showed an improvement in cardiac function in *α*MHC.IGF-1Ea compared to WT mice one month after MI [25]. To pinpoint the start of functional improvement we extended this analysis and performed echocardiography before and 1, 3, 5, 7, and 28 days after MI (Supplementary Table 1). One day following MI, both groups displayed a reduction in ejection fraction (EF); however, *α*MHC.IGF-1Ea hearts showed significant improvement in left ventricular EF by day 7 after MI (Figure 1(b) and Supplementary Table 1). Left ventricular end systolic/diastolic volumes significantly increased after MI in WT hearts, indicating left ventricular dilation, while the *α*MHC.IGF-1Ea hearts did not display any such signs (Figure 1(c) and Supplementary Table 1). In support of the functional data, the expression of molecular markers for cardiac damage such as actin-alpha 1 skeletal muscle (ACTA) and atrial natriuretic peptide (ANP) was significantly reduced in *α*MHC.IGF-1Ea compared to WT mice 28 days after MI (Figures 1(d) and 1(e)). The peak of endogenous IGF-1Ea in the ischemic region of WT mice by day 7, along with the improvement in cardiac function in *α*MHC.IGF-1Ea as early as day 7, indicates that IGF-1Ea signalling at early time points is key for cardiac repair.

### 3.2. Altered Tissue Remodelling in *α*MHC.IGF-1Ea after Injury

**Figure 2:**
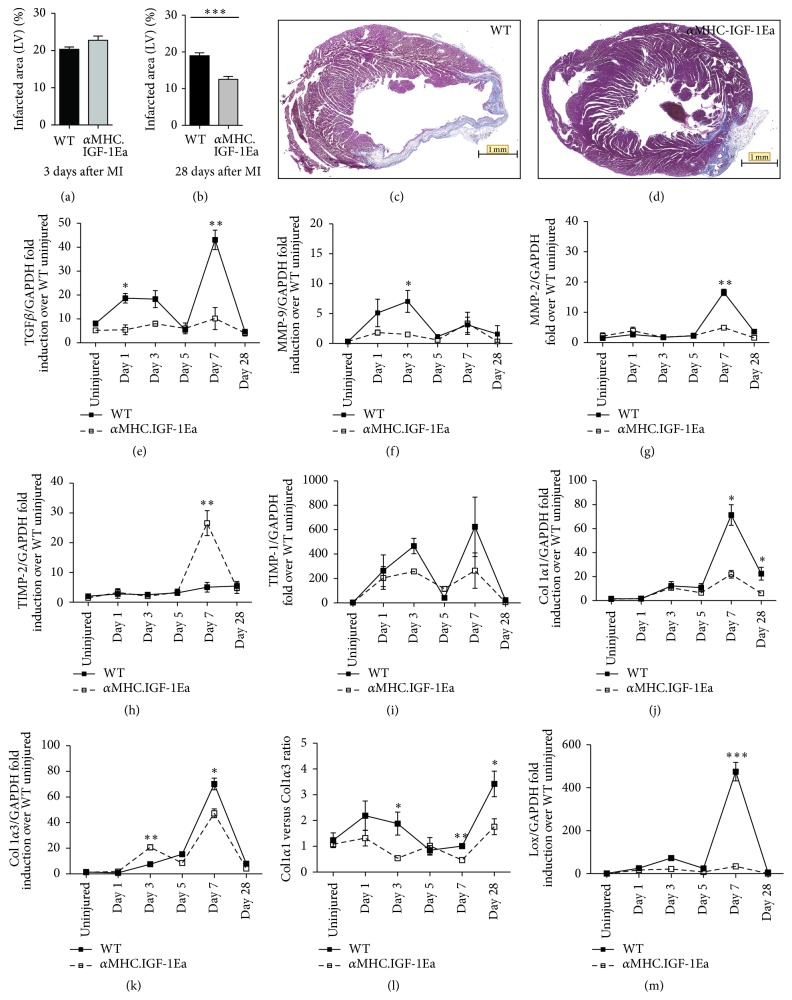
Positive remodeling in the *α*MHC.IGF-1Ea infarcted region after myocardial infarction. Quantification of the infarcted area at (a) 3 days and (b) 28 days after MI. *n* = 3. Representative histological sections of (c) WT and (d) *α*MHC.IGF-1Ea hearts stained with Masson's trichrome 28 days after MI. Scale bar: 1 mm. ((e)–(l)) mRNA relative expression of (e) transforming growth factor beta (TGF-*β*), (f) matrix metalloproteinase 9 (MMP-9), (g) matrix metalloproteinase 2 (MMP-2), (h) tissue inhibitor of metalloproteinase (TIMP) 2, (i) TIMP-1, (j) collagen (Col) I*α*1, (k) Col I*α*3, (l) ColI*α*1/ColI*α*3 ratio, (m) lysyl oxidase in the infarct area. Results are expressed as mean fold induction ± SEM. Solid lines represent WT mice. Dashed lines represent *α*MHC.IGF-1Ea mice. *n* = 3 per group. Two-tailed Student's* t*-test was performed to compare WT versus *α*MHC.IGF-1Ea at selected time points after MI. ^*∗*^ *P* < 0.05, ^*∗∗*^ *P* < 0.005, ^*∗∗∗*^ *P* < 0.001.

Three days after MI, WT and *α*MHC.IGF-1Ea hearts displayed infarcts of equal size, quantified by Masson's trichrome staining (Figure 2(a)). However, by 28 days after infarction, *α*MHC.IGF-1Ea hearts exhibited smaller scar areas compared to WT (Figures 2(b), 2(c) and 2(d)), as previously reported [25]. This was observed as a reduction in scar length but increased scar thickness, consistent with reduced infarct expansion. As a molecular measurement of fibrosis, we quantified TGF-*β* mRNA levels which were significantly lower in *α*MHC.IGF-1Ea than in WT mice after MI (Figure 2(e)). We therefore measured expression of genes involved in ECM turnover and synthesis, MMP-2, MMP-9, TIMP-1, TIMP-2, Col I*α*1, Col I*α*3, and lysyl oxidase (Lox) at 1, 3, 5, 7, and 28 days after MI. In WT hearts, MMP-9 was upregulated 3 days after MI, followed by MMP-2 at day 7 (Figures 2(f) and 2(g)). Interestingly, neither MMP-2 nor MMP-9 was significantly upregulated in *α*MHC.IGF-1Ea hearts at any time point. Inhibitors of matrix degradation, TIMP-1 and TIMP-2, had similar expression in WT and *α*MHC.IGF-1Ea hearts at all time points except for day 7 when TIMP-2 was significantly upregulated in *α*MHC.IGF-1Ea hearts compared to WT (Figures 2(h) and 2(i)). Taken together, this supports the idea of a net overall reduction in matrix breakdown, which may contribute to the reduced fibrosis observed in *α*MHC.IGF-1Ea hearts.

We next analysed the composition of the newly synthesised matrix by measuring mRNA expression of the most abundant cardiac ECM collagen, Col I*α*1 and Col I*α*3, as well as the collagen cross-linker, lox, which increases matrix stiffness. Upregulation of both collagen types was detected by day 3, peaking at day 7 (Figures 2(j) and 2(k)). At this time point, WT hearts expressed significantly more of both collagen types than *α*MHC.IGF-1Ea hearts. We also noted a difference in the ratio of the two collagen types; *α*MHC.IGF-1Ea hearts had a reduced Col I*α*1/Col I*α*3 ratio compared to WT, which was significant at days 3, 7, and 28 (Figure 2(l)). At the peak of collagen upregulation, lox was also upregulated in WT hearts, yet this was not observed in *α*MHC.IGF-1Ea hearts (Figure 2(m)). These results indicate that IGF-1Ea overexpression reduces ECM turnover after MI and alters the composition of the matrix, with preferential expression of Col I*α*3 over Col I*α*1 and less cross-linking, which likely alters the mechanical properties of the fibrotic area.

### 3.3. Distinct Chemokine and Cytokine Production in *α*MHC-IGF-1Ea Hearts after Myocardial Infarct

**Figure 3:**
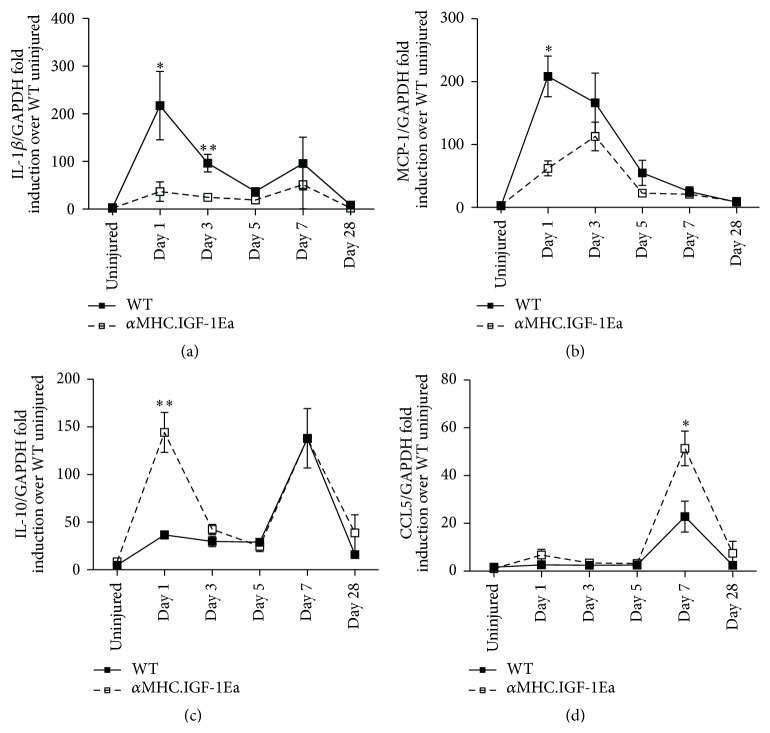
Cytokine dynamics in the infarcted *α*MHC.IGF-1Ea hearts. (a) Interleukin- (IL-) 1*β*, (b) MCP-1, (c) IL-10, (d) CCL5 at several time points after permanent ligation. Solid lines represent WT mice. Dashed lines represent *α*MHC.IGF-1Ea mice. Results are expressed as mean fold induction ± SEM over the values of uninjured hearts. *n* = 3 per group. Two-tailed Student's* t*-test was performed to compare WT versus *α*MHC.IGF-1Ea at selected time points after MI. ^*∗*^ *P* < 0.05, ^*∗∗*^ *P* < 0.005.

Tissue remodelling after injury is closely tied to the inflammatory process. We therefore compared the inflammatory status of the *α*MHC.IGF-1Ea and WT hearts, monitoring the production of key immune genes IL-1*β*, IL-10, MCP-1, and CCL5. Expression of the inflammatory cytokine IL-1*β* was rapidly induced upon injury in WT hearts (66-fold over uninjured) yet was not upregulated as strongly in *α*MHC.IGF-1Ea hearts (19-fold over uninjured; Figure 3(a)). Similarly the strong upregulation of MCP-1 in WT hearts was not observed in *α*MHC.IGF-1Ea hearts (Figure 3(b)). IL-10 was also rapidly upregulated, peaking 1 day after MI. In contrast to IL-1*β* and MCP-1, this immunosuppressive cytokine was upregulated 3-fold higher in *α*MHC.IGF-1Ea hearts compared to WT controls. Although a second peak of IL-10 mRNA at 7 days was comparable in both WT and *α*MHC.IGF-1Ea hearts 7 days after MI (Figure 3(c)). CCL5, which is involved in the recruitment of Ly6C^low^ monocytes [26, 27], was upregulated in *α*MHC.IGF-1Ea hearts 7 days after MI (Figure 3(d)). These data suggest an early bias towards a less inflammatory environment potentially modulating the recruitment of monocytes in *α*MHC.IGF-1Ea hearts after myocardial infraction.

### 3.4. IGF-1Ea Modulates Myeloid Cell Recruitment after Myocardial Infarction

To document accumulation of the main innate immune cell populations involved in cardiac inflammation after infarct, single cell suspensions were prepared from whole mouse hearts and analysed by flow cytometry. All cell populations described in this work were identified using the gating strategy shown in Supplementary Figures  3–6.

**Figure 4:**
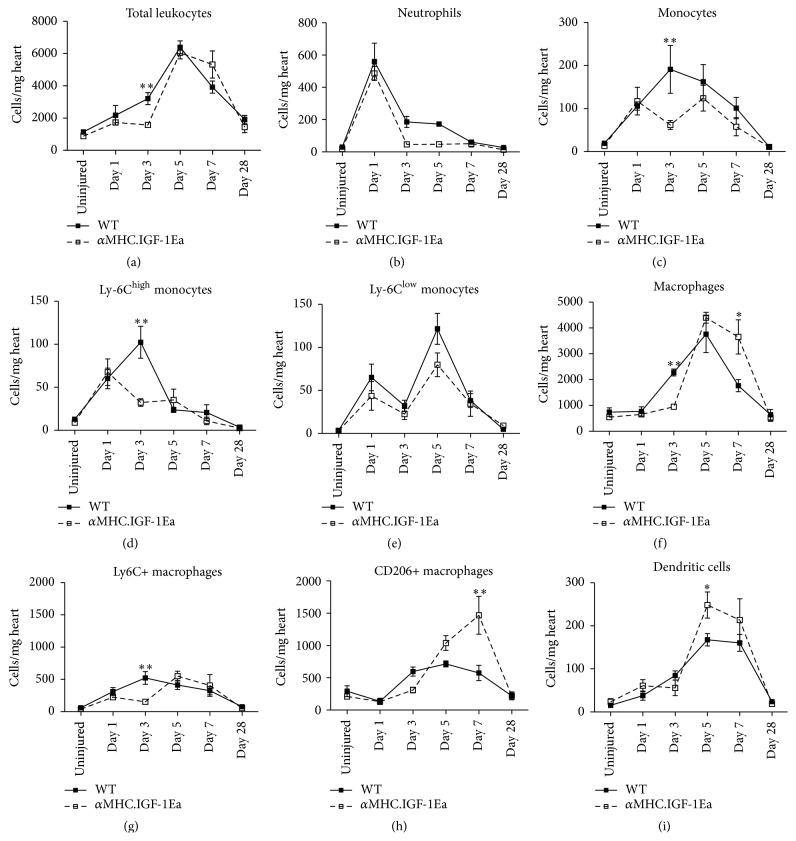
Characterisation and temporal dynamics of immune cell populations in *α*MHC.IGF-1Ea hearts after MI. Quantification of (a) total leukocytes (CD45+), (b) neutrophils (CD45+, CD11b+, F4/80−, CD11c−, and Ly6G+), and (c) monocytes (CD45+, CD11b+, CD11c−, Ly6G−, and F4/80−) which were further classified as (d) Ly6C^high^ and (e) Ly6C^low^ monocytes. (f) Macrophages (CD45+, CD11b+, CD11c−, Ly6G−, and F4/80+) were further characterised on the basis of Ly6C and CD206 expression as (g) Ly6C^high^/CD206^low^ (inflammatory macrophages) and (h) Ly6C^low^/CD206^high^ (reparative macrophages). For this work, only cells that were either Ly6C^high^/CD206− or Ly6C−/CD206+ were analysed, although we noted a double positive cell population (i.e., CD206+, Ly6C+). (i) Dendritic cells were defined as CD45+, CD11b+, F4/80−, CD11c+, and Ly6G−. Data is presented as the total number of cells per mg of heart. *n* = 4–6 per group. Two-tailed Student's* t*-test was performed to compare WT versus *α*MHC.IGF-1Ea at selected time points after MI. ^*∗*^ *P* < 0.05, ^*∗∗*^ *P* < 0.005.

In WT and *α*MHC.IGF-1Ea hearts, the total number of infiltrating leukocytes (CD45+) gradually increased after MI, both reaching comparable peak numbers at day 5 (Figure 4(a)). However at the earlier 3-day time point, *α*MHC.IGF-1Ea hearts contained 49% less leukocytes per milligram of tissue than WT hearts. In examining specific immune cell populations, this difference was partly explained by a 75% reduction in neutrophils (CD45+, CD11b+, F4/80−, CD11c−, and Ly6G+; 186 versus 46 cells/mg of tissue, Figure 4(b)) and a 67% reduction in monocytes (CD45+, CD11b+, CD11c−, Ly6G−, and F4/80−; 191 versus 62 cells/mg of heart, Figure 4(c)); however it was mostly due to reduced presence of macrophages (CD45+, CD11b+, CD11c−, Ly6G−, and F4/80+; 58%, 2276 versus 949 cells/mg of heart, Figure 4(f)). These data agree with the reduced MCP-1 expression observed in *α*MHC.IGF-1Ea hearts (Figure 3(b)), as it is the principal chemokine involved in monocyte recruitment [26, 28].

The Ly6C surface marker distinguishes two different subsets of monocytes [2, 3]. Analysis of the Ly6C^high^ (CD45+, CD11b+, CD11c−, Ly6G−, F4/80−, and Ly6C^high^) and Ly6C^low^ (CD45+, CD11b+, CD11c−, Ly6G−, F4/80−, and Ly6C^low^) populations revealed that the reduction of total monocyte numbers at day 3 was attributable to a 20% decrease of the Ly6C^high^ population (102 versus 32 cells/mg of heart), whereas the Ly6C^low^ population was not significantly different in WT compared to *α*MHC.IGF-1Ea (Figures 4(d) and 4(e)) hearts.

To differentiate between inflammatory and reparative macrophage populations, we used the markers Ly6C and CD206. For this work, only cells that were either Ly6C+/CD206− or Ly6C−/CD206+ were analysed, although we noted a double positive cell population (i.e., CD206+, Ly6C+). However no changes were observed over time between WT and *α*MHC.IGF-1Ea for the double positive population (Supplementary 2). Both Ly6C+ inflammatory macrophage (CD45+, CD11b+, CD11c−, Ly6G−, F4/80+, and Ly6C+/CD206−) and CD206+ reparative macrophage (CD45+, CD11b+, CD11c−, Ly6G−, F4/80+, and Ly6C−/CD206+) normalised cell numbers were reduced by 71% and 48%, respectively, in *α*MHC.IGF-1Ea hearts at the day 3 time point (522 versus 153 and 596 versus 310 cells/mg of tissue of heart, resp.; Figures 4(g) and 4(h)); however this was significant only for the Ly6C+ population. By day 7 after MI, macrophage dynamics changed and we observed a greater number of total macrophages in *α*MHC.IGF-1Ea hearts compared to WT, which was mainly due to a 155% preferential increase in the CD206+ population (575 versus 1468 cells/mg heart, Figure 4(h)). Dendritic cells (CD45+, CD11b+, F4/80−, CD11c+, and Ly6G−) were increased by 48% in *α*MHC.IGF-1Ea hearts compared to WT 5 days after MI (168 versus 248 cells/mg heart; Figure 4(i)).

In summary, cardiac-restricted expression of an IGF-1Ea transgene limited the early accumulation of innate immune cells at day 3 after MI, with a bias towards the reduction of inflammatory myeloid populations rather than regenerative populations. This reduction corresponds with the lower expression of myeloid chemokines and the less inflammatory milieu observed in the *α*MHC.IGF-1Ea hearts.

## 4. Discussion

Previous work in our lab showed that local expression of IGF-1Ea promoted functional restoration after MI and observed reduced infarct expansion, thinning, and dilation of the left ventricular wall [25]. We now demonstrate transcriptional modulation of key ECM remodelling genes in the IGF-1Ea hearts associated with tempering of the inflammatory myeloid cell response.

Increased MMP expression after injury has been implicated in contributing to scar destabilisation, as transgenic animal model knockouts of either MMP-2 or MMP-9 have been shown to attenuate LV dilation, rupture, and impairment of cardiac function [29, 30]. While MMP-2 and MMP-9 mRNA expression levels were upregulated in injured WT hearts, this increase was not observed in *α*MHC.IGF-1Ea hearts. In line with reduced matrix breakdown, mRNA expression of the MMP inhibitor, TIMP-2, was significantly increased in *α*MHC.IGF-1Ea hearts at day 7 after MI. Thus IGF-1Ea may prevent adverse cardiac remodelling, in part, by modulating transcription of MMPs/TIMPs.

The production of new matrix components was also altered by the presence of the IGF-1Ea transgene with an overall reduction in collagen synthesis, confirmed in histological stains, and a bias towards expression of Col I*α*3 over Col I*α*1. In MI patients, turnover of cardiac extracellular matrix can be assessed by using circulating collagen peptides as blood biomarkers [31] and high type I collagen is associated with adverse clinical outcome [32]. A prospective multicentre study further showed that a low type III/type I collagen ratio especially at 1 month after MI is predictive of detrimental left ventricular remodelling as well as cardiovascular deaths and hospitalisation cases for heart failure [33]. Changes in collagen ratios are known to affect the strength and tensile properties of the ECM. It would be interesting to determine whether these properties are modified in the *α*MHC.IGF-1Ea hearts.

IGF-1Ea could promote the changes in collagen deposition by directly acting on fibroblasts as it promotes both their proliferation and activation to myofibroblasts [34, 35]. Alternatively, IGF-1Ea may influence accumulation and activation of immune cells present at the infarct which in turn regulate myofibroblast activation. Indeed, we observed modulation of the inflammatory process in *α*MHC.IGF-1Ea hearts, with less monocyte (Ly6C^high^) infiltration into the injured myocardium. This effect is associated with reduced expression of the monocyte chemoattractant MCP-1, although no significant changes were observed in the Ly6C^low^ monocyte population in infarcted hearts, possibly due to CCL5 upregulation.

It is interesting that while complete depletion of monocytes at any stage of repair leads to poor recovery [36], a more subtle modulation of the monocyte population in the *α*MHC.IGF-1Ea hearts is associated with improved heart function. Studies abrogating monocyte recruitment using a selective CCR2 inhibitor have resulted in reduced IL-1*β*, IL-6, MCP-1, and TNF*α* levels [37]. IGF-1Ea influences macrophage polarisation in skeletal muscle [38] and we similarly found reduced Ly6C^high^ monocyte and Ly6C+ macrophage normalised cell numbers while CD206+ macrophage numbers were increased by day 7, suggesting that IGF-1Ea promoted a quick progression to the reparative phase of repair by modulating macrophage phenotype. In the *α*MHC.IGF-1Ea hearts we observed a decrease in MCP-1, which is the chemokine for CCR2, recently shown to distinguish infiltrating monocytes from the resident macrophage population which has a different embryological origin and expresses CD206 [39–41]. It is therefore possible that, in our *α*MHC.IGF-1Ea transgenic mouse model, IGF-1Ea reduces the infiltration of inflammatory Ly6C^high^ monocytes by preventing upregulation of MCP-1 while still allowing for expansion of the resident macrophage population.

In summary, we show that the *α*MHC.IGF-1Ea mouse model can modulate several associated aspects of the cellular repair process after MI, including immune cell recruitment, cytokine expression, and matrix turnover. All of these changes occur within the first 7 days after infarct, at which time a functional improvement can already be measured in *α*MHC.IGF-1Ea hearts compared to WT controls. These data provide new insights into the mechanism of IGF-1Ea and suggest that early immunomodulation is key to successful cardiac repair after injury.

## Supplementary Material

Supplementary 1. IGF-1Ea relative levels in the heart after myocardial infarct.
Supplementary 2. Double CD206+/Ly6C+ population.
Supplementary 3. Gating strategy for infiltrating immune cells after myocardial infarct.
Supplementary 4. Fluorescence minus one (FMO) controls plots for Ly6G and CD11c. Based on the gating strategy in Supplementary figure 3, FMO controls were used to set the threshold gate. Single cell suspensions isolated from hearts of mice uninjured and post-MI were stained with anti-CD11b, -CD45, –Ly-6G, –Ly-6C, -F4/80, -CD206 -CD11c except for the fluorochrome being negatively gated. For the Ly6G FMO control, single cell suspensions isolated from hearts of mice uninjured and post-MI were stained with all the fluorochromes except Ly6G. For the CD11c FMO controls single cell suspensions isolated from hearts of mice uninjured and post-MI were stained with all the fluorochromes except CD11c.
Supplementary 5. Fluorescence minus one (FMO) controls plots for CD11b and F4/80. Based on the gating strategy in Supplementary figure 3, FMO controls were used to set the threshold gate. Single cell suspensions isolated from hearts of mice uninjured and post-MI were stained with anti-CD11b, -CD45, –Ly-6G, –Ly-6C, -F4/80, -CD206 -CD11c except for the fluorochrome being negatively gated. For the CD11b FMO control, single cell suspensions isolated from hearts of mice uninjured and post-MI were stained with all the fluorochromes except CD11b. For the F4/80 FMO controls single cell suspensions isolated from hearts of mice uninjured and post-MI were stained with all the fluorochromes except F4/80.
Supplementary 6. Fluorescence minus one (FMO) controls plots for Ly6C and CD206. Based on the gating strategy in Supplementary figure 3, FMO controls were used to set the threshold gate. Single cell suspensions isolated from hearts of mice uninjured and post-MI were stained with anti-CD11b, -CD45, –Ly-6G, –Ly-6C, -F4/80, -CD206 -CD11c except for the fluorochrome being negatively gated. For the Ly6C FMO control, single cell suspensions isolated from hearts of mice uninjured and post-MI were stained with all the fluorochromes except Ly6C. For the CD206 FMO controls single cell suspensions isolated from hearts of mice uninjured and post-MI were stained with all the fluorochromes except CD206.
Supplementary table 1. Echocardiographic measurements and analysis performed at 1, 3, 5, 7 and 28 days after MI.
