# Supplementary material for: Cardiac-Restricted IGF-1Ea Overexpression Reduces the Early Accumulation of Inflammatory Myeloid Cells and Mediates Expression of Extracellular Matrix Remodelling Genes after Myocardial Infarction
Source: Mediators Inflamm. 2015 Sep 30;2015:484357. doi: 10.1155/2015/484357 (PMC4605352; doi:10.1155/2015/484357)
Supplement: Supplementary file 1 — Supplementary 1. IGF-1Ea relative levels in the heart after myocardial infarct. Supplementary 2. Double CD206+/Ly6C+ population. Supplementary 3. Gating strategy for infiltrating immune cells after myocardial infarct. Supplementary 4. Fluorescence minus one (FMO) controls plots for Ly6G and CD11c. Based on the gating strategy in Supplementary figure 3, FMO controls were used to set the threshold gate. Single cell suspensions isolated from hearts of mice uninjured and post-MI were stained with anti-CD11b, -CD45, –Ly-6G, –Ly-6C, -F4/80, -CD206 -CD11c except for the fluorochrome being negatively gated. For the Ly6G FMO control, single cell suspensions isolated from hearts of mice uninjured and post-MI were stained with all the fluorochromes except Ly6G. For the CD11c FMO controls single cell suspensions isolated from hearts of mice uninjured and post-MI were stained with all the fluorochromes except CD11c. Supplementary 5. Fluorescence minus one (FMO) controls plots for CD11b and F4/80. Based on the gating strategy in Supplementary figure 3, FMO controls were used to set the threshold gate. Single cell suspensions isolated from hearts of mice uninjured and post-MI were stained with anti-CD11b, -CD45, –Ly-6G, –Ly-6C, -F4/80, -CD206 -CD11c except for the fluorochrome being negatively gated. For the CD11b FMO control, single cell suspensions isolated from hearts of mice uninjured and post-MI were stained with all the fluorochromes except CD11b. For the F4/80 FMO controls single cell suspensions isolated from hearts of mice uninjured and post-MI were stained with all the fluorochromes except F4/80. Supplementary 6. Fluorescence minus one (FMO) controls plots for Ly6C and CD206. Based on the gating strategy in Supplementary figure 3, FMO controls were used to set the threshold gate. Single cell suspensions isolated from hearts of mice uninjured and post-MI were stained with anti-CD11b, -CD45, –Ly-6G, –Ly-6C, -F4/80, -CD206 -CD11c except for the fluorochrome being negatively [file 484357.f1.pdf]

Supplementary 1.

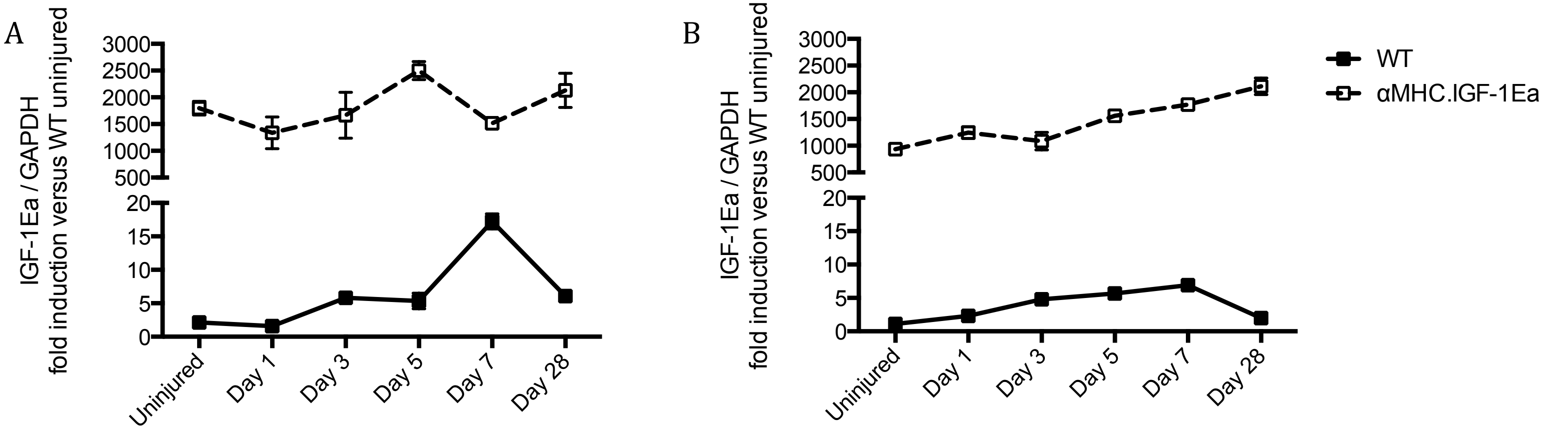

Supplementary 2.

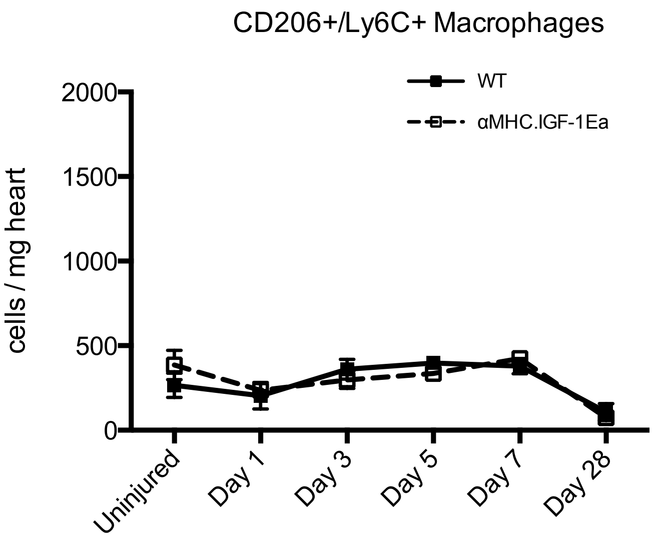

Supplementary 3.

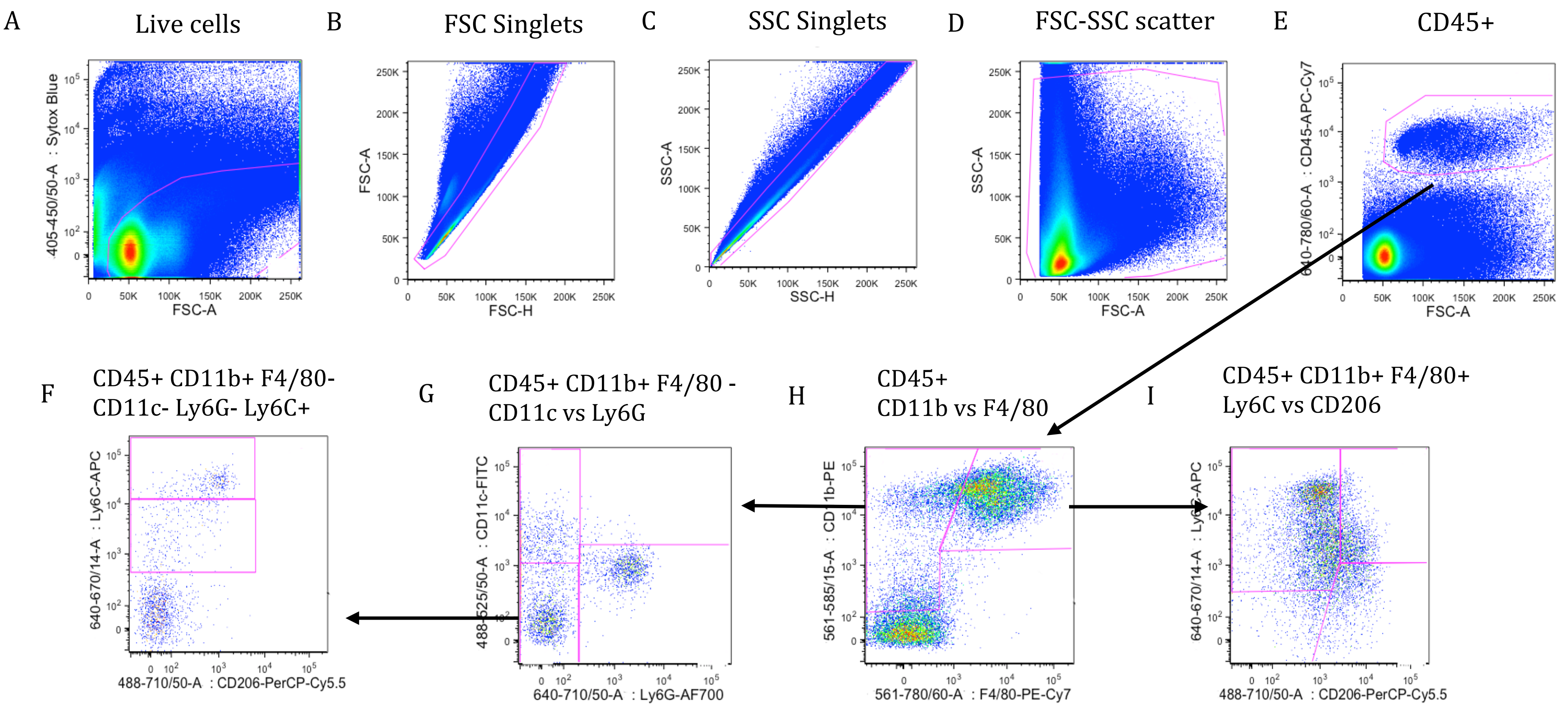

Supplementary 4.

FMO#1 – no Ly6G

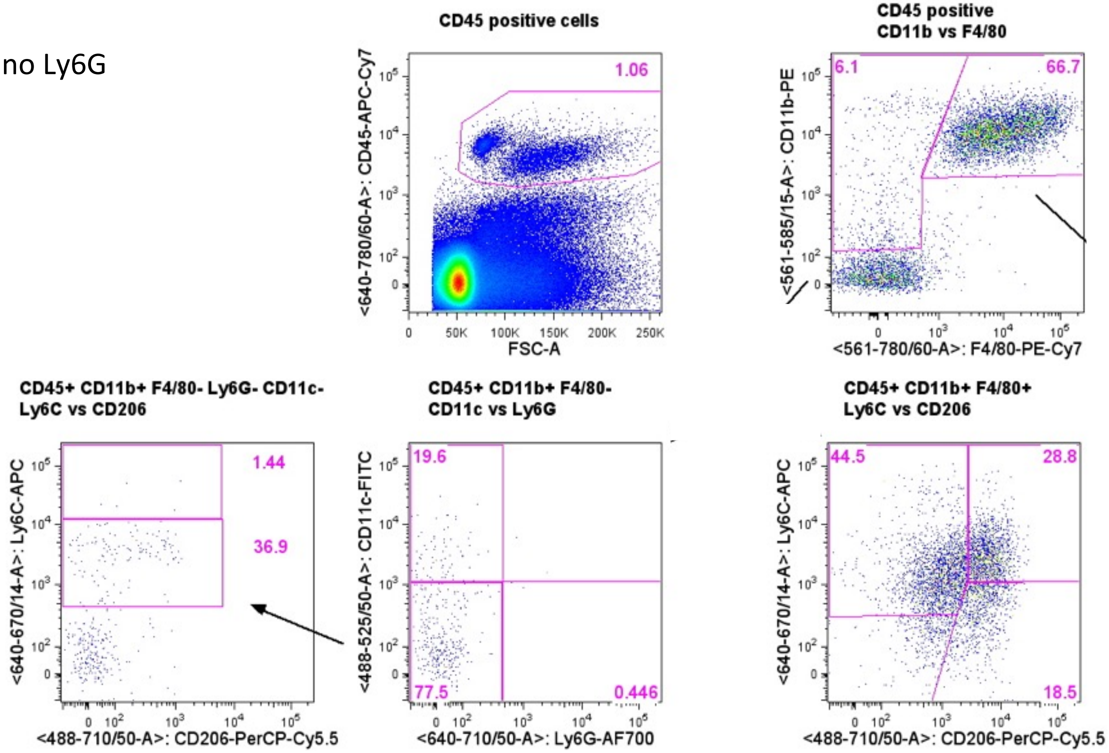

FMO#2 – no CD11c

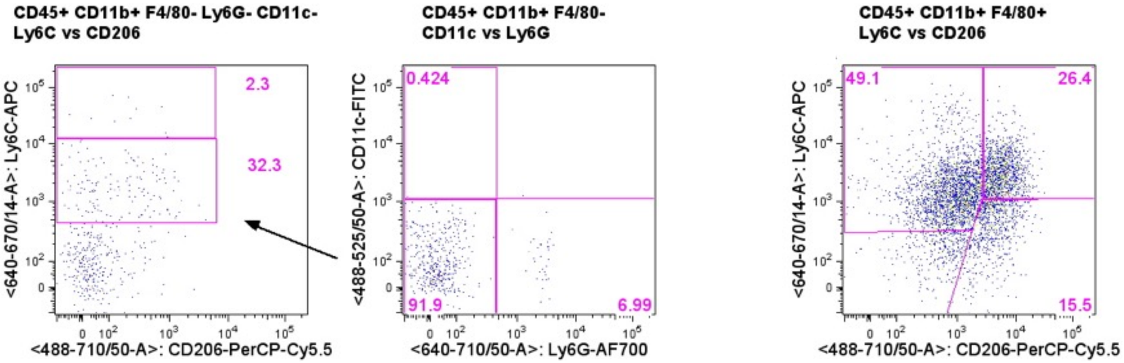

Supplementary 5.

FMO#3 – no CD11b

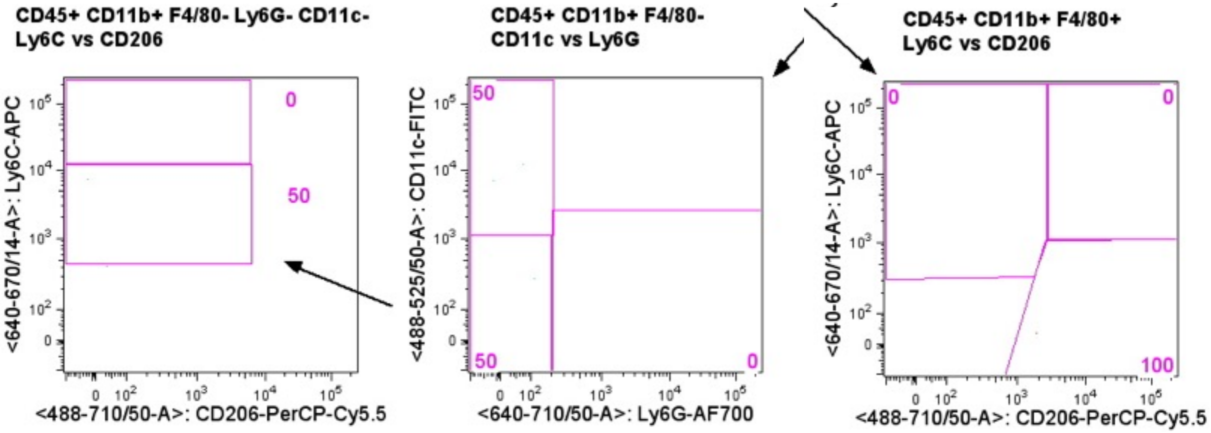

FMO#4 – no F4/80

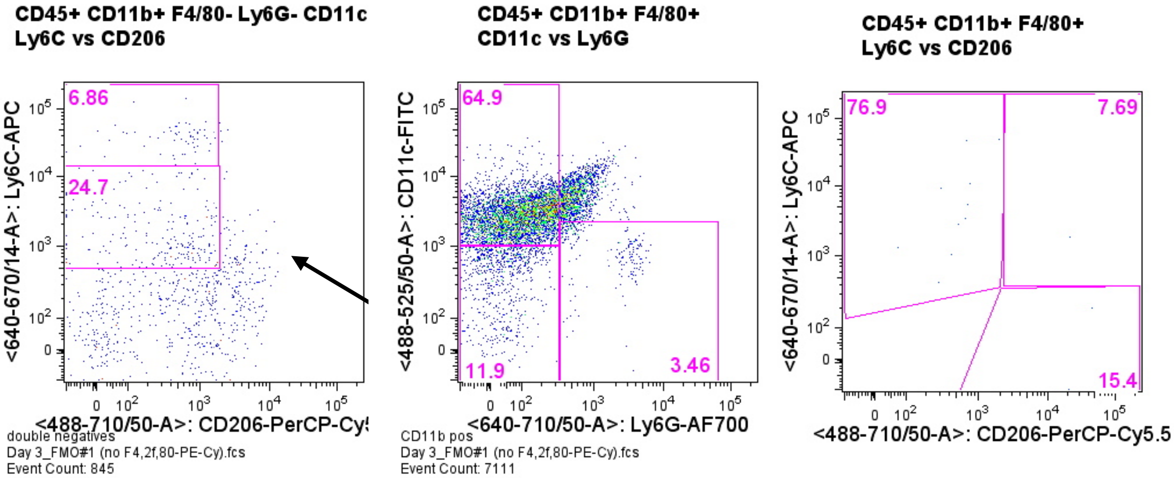

Supplementary 6.

FMO#5 – no Ly6C

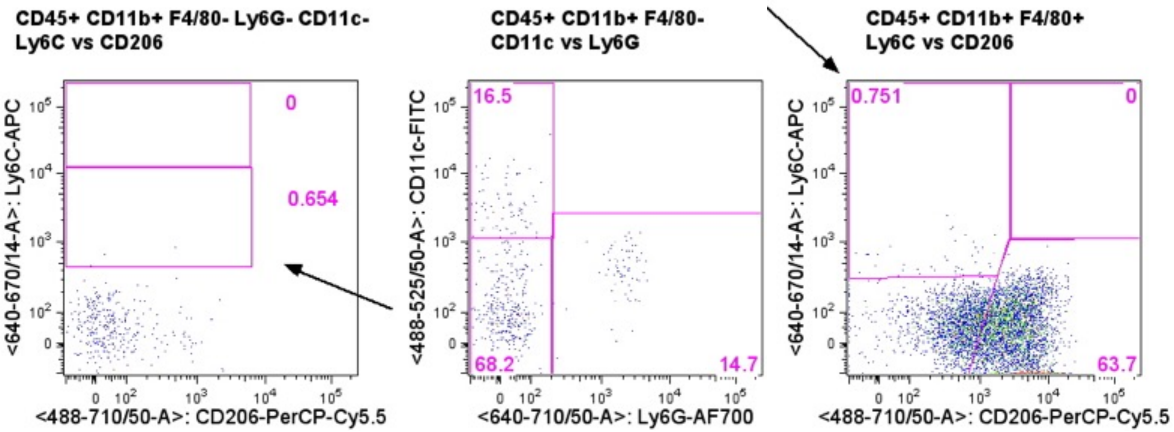

FMO#6 – no CD206

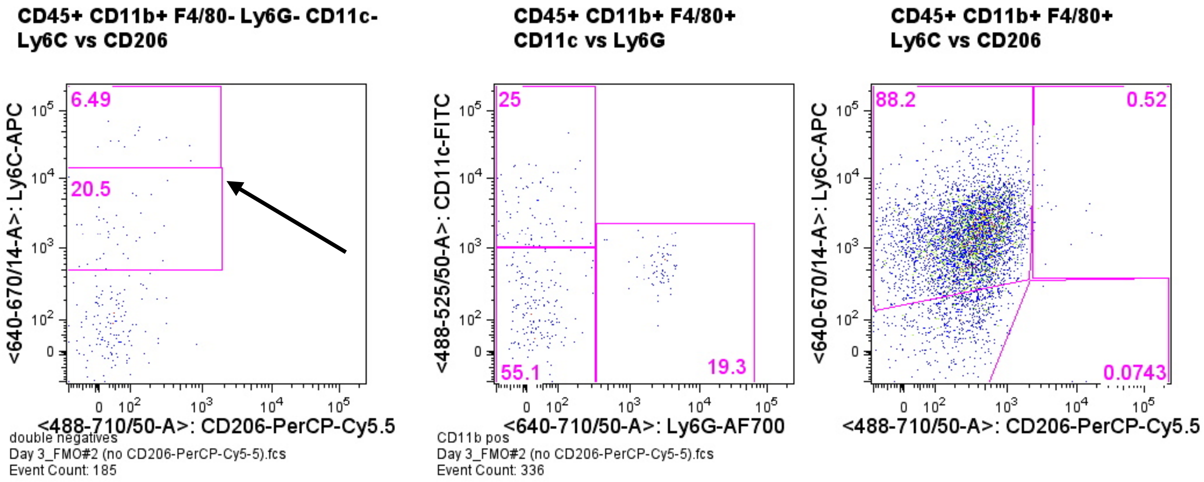

Supplementary table 1.

	WT	αMHC-IGF-1Ea	WT	αMHC-IGF-1Ea
	Uninjured		1 Day	
**LVEDVd**	52,82±5,246	70,05±10,54	55,09±8,99	67,83±7,35
**LVEDVs**	18,91±3,93	32,79±9,10	39,31±9,35	44,57±12,51
**LVIDd**	3,71±0,26	4,08±0,18	3,44±0,29	3,75±0,30
**LVIDs**	2,47±0.182	3,01±0,29	3,05±0,52	3,24±0,40
**LVPWd**	*0,83±0,33*	1,02±0,07	0,95±0,07	0,85±0,05
**LVPWs**	1,10±0,071	1,18±0,11	1,07±0,14	0,98±0,19
**LVEF**	64,652±2,72	67,57±3,21	37,24±8,11&	29,96±9,266&
**%FAC**	38,80±1,91	37,43±2,36	18,83±4,35&	23,52±7,91
	3 Days		5 Days	
**LVEDVd**	57,13±5,271	57,14±4,26	75,610±15,68	59,930±10,355
**LVEDVs**	*34,97±4,01*	26,48±3,14	50,66±6,51	34,82±9,22
**LVIDd**	*3,78±0,07*	3,59±0,452	4,06±0,427	3,69±0,24
**LVIDs**	*2,92±0.15*	2,75±0,31	3,11±0,33	2,99±0,36
**LVPWd**	*0,86±0,08*	0,74±0,04	0,67±0,09	0,88±0,17
**LVPWs**	*1,00±0,09*	0,94±0,25	0,70±0,08	1,09±0,18
**LVEF**	43,72±2,851&	54,17±2,80	34,80±4,24&	46,51±7,95
**%FAC**	*22,51±2,27*	28,90±3,11	16,60±2,34&	23,43±4,59&
	7 Days		1 Month	
**LVEDVd**	78,95±14,71	56,88±11,5	101,88±20,54&	63,29±6,34
**LVEDVs**	*76,61±10,81*&	27,133±5,793*	73,72±14,80&	29,08±3,37*
**LVIDd**	*4,31±0,32*	3,63±0,35	4,38±0,31	3,80±0,16
**LVIDs**	*4,26±0,21*&	2,88±0,53*	3,85±0,27&	2,65±0,14*
**LVPWd**	0,62±0,03	0,70±0,07	0,71±0,05	0,80±0,06
**LVPWs**	*0,60±0,03*	0,96±0,96	0,65±0,03	1,05±0,11*
**LVEF**	24,35±6,52&	53,16±10,24*	35,24±3,38&	59,244±3,949*
**%FAC**	16,69±4,106&	36,340±3,31*	15,636±2,06&	32,783±2,38*
